# Gender-Specific Expression of Ubiquitin-Specific Peptidase 9 Modulates Tau Expression and Phosphorylation: Possible Implications for Tauopathies

**DOI:** 10.1007/s12035-016-0299-z

**Published:** 2016-11-23

**Authors:** Sandra Köglsberger, Maria Lorena Cordero-Maldonado, Paul Antony, Julia Ilona Forster, Pierre Garcia, Manuel Buttini, Alexander Crawford, Enrico Glaab

**Affiliations:** Luxembourg Centre for Systems Biomedicine (LCSB), University of Luxembourg, 7 avenue des Hauts Fourneaux, 4362 Esch-sur-Alzette, Luxembourg

**Keywords:** Transcriptomics, Tau, Tauopathies, Alzheimer’s disease, Gender differences, Zebrafish

## Abstract

Public transcriptomic studies have shown that several genes display pronounced gender differences in their expression in the human brain, which may influence the manifestations and risk for neuronal disorders. Here, we apply a transcriptome-wide analysis to discover genes with gender-specific expression and significant alterations in public postmortem brain tissue from Alzheimer’s disease (AD) patients compared to controls. We identify the sex-linked ubiquitin-specific peptidase 9 (USP9) as an outstanding candidate gene with highly significant expression differences between the genders and male-specific underexpression in AD. Since previous studies have shown that USP9 can modulate the phosphorylation of the AD-associated protein MAPT, we investigate functional associations between USP9 and MAPT in further detail. After observing a high positive correlation between the expression of *USP9* and *MAPT* in the public transcriptomics data, we show that USP9 knockdown results in significantly decreased *MAPT* expression in a DU145 cell culture model and a concentration-dependent decrease for the *MAPT* orthologs *mapta* and *maptb* in a zebrafish model. From the analysis of microarray and qRT-PCR experiments for the knockdown in DU145 cells and prior knowledge from the literature, we derive a data-congruent model for a *USP9*-dependent regulatory mechanism modulating *MAPT* expression via BACH1 and SMAD4. Overall, the analyses suggest *USP9* may contribute to molecular gender differences observed in tauopathies and provide a new target for intervention strategies to modulate MAPT expression.

**Electronic supplementary material:**

The online version of this article (doi:10.1007/s12035-016-0299-z) contains supplementary material, which is available to authorized users.

## Introduction

Gender differences in the incidence and phenotypic manifestations of brain disorders like Alzheimer’s disease (AD) have been reported in several independent studies [1–5]. Findings for AD include significantly more severe pathology in women than in men [1], faster brain atrophy in females [2], as well as a higher AD incidence in females after adjusting for differential survival, which has mainly been observed in the oldest-age categories [3–5].

Some studies have suggested lifestyle differences as a contributing factor for these gender disparities, e.g., the Framingham Heart Study indicated that selective survival of men with a healthier cardiovascular risk profile and hence lower propensity to dementia could partly explain a higher lifetime risk for AD in women [6]. Moreover, hormonal mechanisms have been implicated in AD gender differences, and neuroprotective functions have been proposed for testosterone [7, 8], estrogen [9], and progesterone [10]. However, studies according to which the disease incidence rates in other neurodegenerative diseases show an almost opposite distribution between the genders as compared to AD (e.g., a greater risk for Parkinson’s disease in men than in women [11]), suggest that differences in hormonal neuroprotection without disease specificity are not the only factor influencing the observed gender differences in neurodegenerative disorders. Similarly, more severe AD-associated effects of the ε4 variant of the *APOE* gene, the largest known genetic risk factor for sporadic AD, reported for females [12] cannot account for the entire range of phenotypic differences in the disease between the genders, because cognitive impairment progresses faster in women than in men even when considering only individuals without *APOE* ε4 alleles [13].

Overall, previous studies suggest that a multitude of factors is likely to be involved in AD gender differences, and accordingly, system level analyses of biomolecular differences between the sexes in AD may help to identify and understand further relevant factors. Although gender-based disease alterations in individual biomolecules are unlikely to explain the full spectrum of observed gender-specific disease manifestations, their identification and analysis may provide new pointers to biomolecules with important regulatory functions in the studied disease, supporting the design of new therapeutic interventions.

Since major differences in brain gene expression levels between males and females have previously been reported [14], but not investigated in the context of AD, we have conducted a corresponding system-wide transcriptome analysis on public data. In subsequent experiments for the top candidate gene identified, the *ubiquitin-specific peptidase 9* (*USP9*), we investigated which potential AD disease mediators the gene is linked to. *USP9* is a gonosomal gene with a Y-chromosomal (*USP9Y*) and an X-chromosomal form. In humans, *USP9X* escapes X-inactivation [15]. The gene encodes a deubiquitinase [16], removing the ubiquitin moieties from specific proteins and thereby preventing their proteasomal degradation [15, 17]. It deubiquitinates monoubiquitinated SMAD4 and has been shown to be an essential component of the TGF-beta/BMP signaling cascade [16] (the full gene names and brief function descriptions for USP9, SMAD4, and the other main genes discussed in this manuscript is provided in Table 1).

**Table 1:** Overview of the main genes discussed in this article

Gene symbol	Full gene name	Description (from GeneCards [18])
*USP9X/Y*	Ubiquitin specific peptidase 9, X/Y	Deubiquitinase, preventing degradation of specific proteins through the removal of conjugated ubiquitin
*MAPT*	Microtubule-associated protein tau	Promotes microtubule assembly and stability; found mutated in several neurodegenerative disorders
*BACH1*	BTB and CNC homology 1, basic leucine zipper transcription factor 1	Transcriptional regulator that acts as repressor for *MAPT*
*SMAD4*	SMAD family member 4	Member of the SMAD family of signal transduction proteins, represses transcription of *BACH1*
*MARK4*	MAP/microtubule affinity-regulating kinase 4	Member of the microtubule affinity-regulating kinase family, phosphorylates MAPT
*GSK3B*	Glycogen synthase kinase 3 beta	Serine-threonine kinase, belonging to the glycogen synthase kinase subfamily, phosphorylates MAPT

Full gene names and descriptions of some of the known relevant gene functions are provided (using information from the GeneCards repository)

In this study, we first describe the analyses of public transcriptomics data that led to the identification of *USP9* as a gene with an outstanding gender-linked expression pattern and significant alterations in AD. We then discuss evidence for regulatory links between *USP9* and the AD-associated protein tau (MAPT), derived from previous studies in the literature, as well as *USP9* knockdown experiments we performed in two model systems, a zebrafish model and human DU145 prostate cancer cells. Analyzing the transcriptome alterations in the DU145 cells in response to the knockdown experiments at the level of cellular pathways and molecular networks, we find significant alterations in cytoskeleton remodeling processes and specifically, in the molecular network of microtubule-associated proteins (*MAPs*) and tubulins linked to *MAPT*. From a network analysis of this dataset, we derive a model for the involvement of *USP9* in the regulation of *MAPT* expression and phosphorylation and discuss possible applications for developing USP9-based intervention strategies targeting tau hyperphosphorylation and aggregation in AD and other tauopathies.

## Results and Discussion

### USP9Y Displays Significant Gender-Linked Expression in the Adult Brain and Diminished Expression in Alzheimer’s Disease

**Fig. 1:**
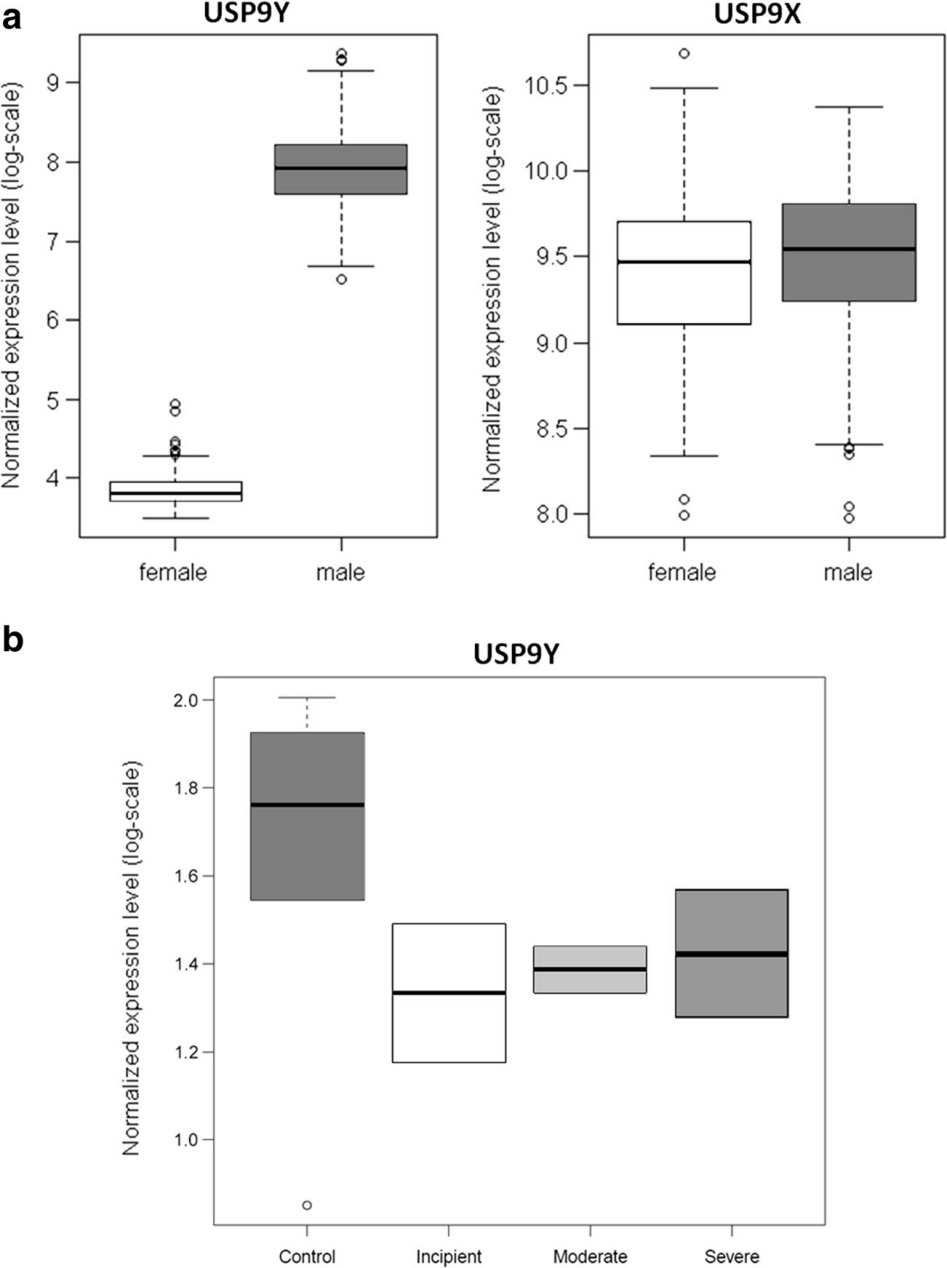
**a** *Left*: *USP9Y* normalized gene expression levels in *postmortem* brain tissue from adult males and females not affected by AD from the HBT dataset (as expected, the signal for the Y-chromosomal version of the gene in female samples does not significantly exceed the baseline, i.e., no expression is detected); *right*: *USP9X* normalized expression levels in *postmortem* brain tissue from adult males and females (expression levels are comparable across the genders, i.e., *USP9X* expression in females does not compensate the absence of *USP9Y* expression in females; see also the comparison across different age groups in Fig. 2c). **b** Boxplot of *USP9Y* gene expression levels in human *postmortem* hippocampus samples for incipient, moderate, and severe cases of male Alzheimer’s disease patients as compared to non-demented male controls (dataset by Blalock et al.). The reader should note that normalized expression level intensities are only comparable across different genes within one study but not across different studies

To investigate gender differences in the human brain transcriptome during adulthood which may contribute to sex differences observed in AD, we mined large-scale public datasets for genes that displayed both a generic gender-linked expression and significant expression alterations in AD. For this purpose, first, we computed male to female median gene expression level ratios using *postmortem* microarray data from a subset of 404 samples from 16 different brain regions across all unaffected adult individuals in the Human Brain Transcriptome Project (HBT) [14] (NCBI GEO dataset series GSE25219; the covered brain regions include orbital, dorsolateral, ventrolateral, and medial regions of the prefrontal cortex, primary motor cortex, primary somatosensory cortex, posterior inferior parietal cortex, primary auditory cortex, superior and inferior temporal cortex, primary visual cortex, hippocampus, amygdala, striatum, mediodorsal nucleus of the thalamus, and cerebellar cortex; see the “Methods” section for details on data preprocessing and analysis). As expected, the highest consistent male/female expression ratios across the 16 brain regions were observed for sex-linked genes, the top five being (ordered by decreasing ratio): *PCDH11Y*, *RPS4Y1*, *DDX3Y*, *USP9Y*, and *EIF1AY* (see Fig. 1a, showing a boxplot for *USP9Y* and *USP9X* as an example).

Next, we used data from an independent late-onset AD case/control transcriptomics study [19] (NCBI GEO dataset series GSE44772), covering brain samples from 690 individuals across three brain regions (prefrontal cortex, visual cortex, and cerebellum), to examine which of the identified genes with significant gender-linked expression in the adult human brain also displayed significant and consistent gender-specific expression alterations in AD across the covered brain regions. The rationale is that genes with robust expression alterations across multiple brain regions in a single study can be compared qualitatively with gene expression changes observed in other studies covering the same or further brain regions, to accumulate evidence for consistent multiregional alteration patterns. Among the identified genes with consistent multiregional gender-linked expression as well as gender-specific expression alterations in AD, the deubiquitinase *USP9Y* was the only gene showing a significantly reduced expression in male AD patients as compared to unaffected male subjects (adjusted *p* value <0.05) that matched with the multiregional changes observed on data from another AD case/control study with 161 microarray samples (*p* = 0.003) [20] (NCBI GEO dataset series GSE5281, covering the brain regions primary visual cortex, entorhinal cortex, hippocampus, medial temporal gyrus, posterior cingulated, and superior frontal gyrus) as well as a further AD case/control study with 30 hippocampal microarray samples [21] (NCBI GEO dataset series GSE28146; given the small sample size for the individual genders, on this third dataset significance could not be shown independently, *p* = 0.17, but a statistical meta-analysis across all datasets confirmed the significant underexpression across multiple brain regions, *p* < 2E−05). The dataset by Blalock et al. additionally groups AD patients into different stages of progression (“incipient,” “moderate,” and “severe” AD), and in all three stages, a qualitative reduction in median *USP9Y* expression is observed as compared to the control samples (the sample size is however too small to establish significance for the individual progression groups; see boxplot in Fig. 1b). Thus, we decided to focus our subsequent mechanistic investigation on *USP9* gene regulation.

### Associations Between USP9 Expression and the Expression and Phosphorylation of Tau

The AD-related microtubule-associated protein tau (MAPT) is involved in the stabilization of microtubules, which function as intracellular transport path [22]. Aggregations of paired helical filaments (PHFs) of MAPT into neurofibrillary tangles (NFTs) are known as one of the main hallmarks of AD and are commonly considered as neurotoxic but have also been proposed to represent secondary protective effects, since the reduction in microtubule assembly in AD is independent of MAPT abnormalities [23].

Similarly, different roles have been suggested for MAPT phosphorylation in AD, and potential toxic and protective effects have both been described [24, 25]. USP9 is linked mechanistically to MAPT phosphorylation. It has been shown that the X-chromosomal version of USP9 (USP9X) deubiquitinates the microtubule affinity-regulating kinase 4 (MARK4) [26], which in turn is known to phosphorylate MAPT [27]. A significant and strong increase of MARK4 expression and MARK4-MAPT interactions in AD brains, correlating with the Braak stages of the disease, has been found in *postmortem* human brains [28]. In a *drosophila* Alzheimer’s model, overexpression of the USP9 ortholog *faf* was reported to enhance phospho-MAPT-mediated postsynaptic toxicity of amyloid precursor protein (APP)/Aβ-42 [29]. Moreover, USP9X can increase MAPT phosphorylation via a second mechanism, by deubiquitinating the protein alpha-synuclein (SNCA) [30], which functions as a connecting mediator between the glycogen synthase kinase 3β (GSK3B) and MAPT and has been shown to stimulate MAPT phosphorylation via GSK3B in vitro [31]. While our analyses focus on the relations between *USP9* and *MAPT* at the level of transcriptional regulation (see following sections), the prior knowledge on the involvement of USP9 in the regulation of MAPT phosphorylation provides a first line of evidence in support of a close functional relationship between these two genes/proteins.

For the Y-chromosomal version of USP9 (USP9Y), interactions with MARK4 and SNCA as observed for USP9X have not been reported so far, but USP9Y has a high sequence similarity of 93% to USP9X (quantified using the SIAS software tool with default settings, see http://imed.med.ucm.es/Tools, and the canonical protein sequences for UniProt IDs Q93008 and O00507) and is therefore expected to have largely similar structure and function (this is also confirmed by our cell culture model transcriptome analysis, comparing a *USP9X*-specific knockdown with a *USP9X*/*Y* knockdown; see details below).

Apart from previously reported functional relations between USP9 and MAPT at the protein level, the gene expression data analyzed here suggests a further possible association at the transcriptional level, which was analyzed in more detail via knockdown experiments in this study (see following sections). For a shared genetic probe for *MAPT* contained in the transcriptomics datasets by Liang et al. [20] and Blalock et al. [21], high positive correlations with *USP9Y* were found across the male samples (*r* = 0.483, *p* = 2.3E−07 for cases and controls combined, and *r* = 0.376, *p* = 5.5E−03 in controls only for the data by Liang et al.; *r* = 0.425, *p* = 0.169 for cases and controls combined, and *r* = 0.452, *p* = 0.368 in controls only on the small dataset by Blalock et al.). Similarly, among the unaffected male individuals of the older age group (age ≥ 40) in the HBT dataset, a high correlation between *MAPT* and *USP9Y* was observed (*r* = 0.480, *p* = 1.5E−06). Given that a higher correlation was observed in controls as compared to cases on the dataset by Blalock et al., as opposed to a lower correlation in controls compared to cases on the dataset by Liang et al., the results do not suggest that there is a general trend of higher *MAPT*/*USP9Y* correlations in either case or control samples. Generally, for larger sample sizes, improved *p* value significance scores were obtained for the *MAPT*/*USP9Y* correlation (i.e., when combining case and control samples as opposed to studying control samples only and when considering the HBT and Liang et al. datasets as opposed to the smaller Blalock et al. dataset).

### Age-Dependent Expression of MAPT and USP9

**Fig. 2:**
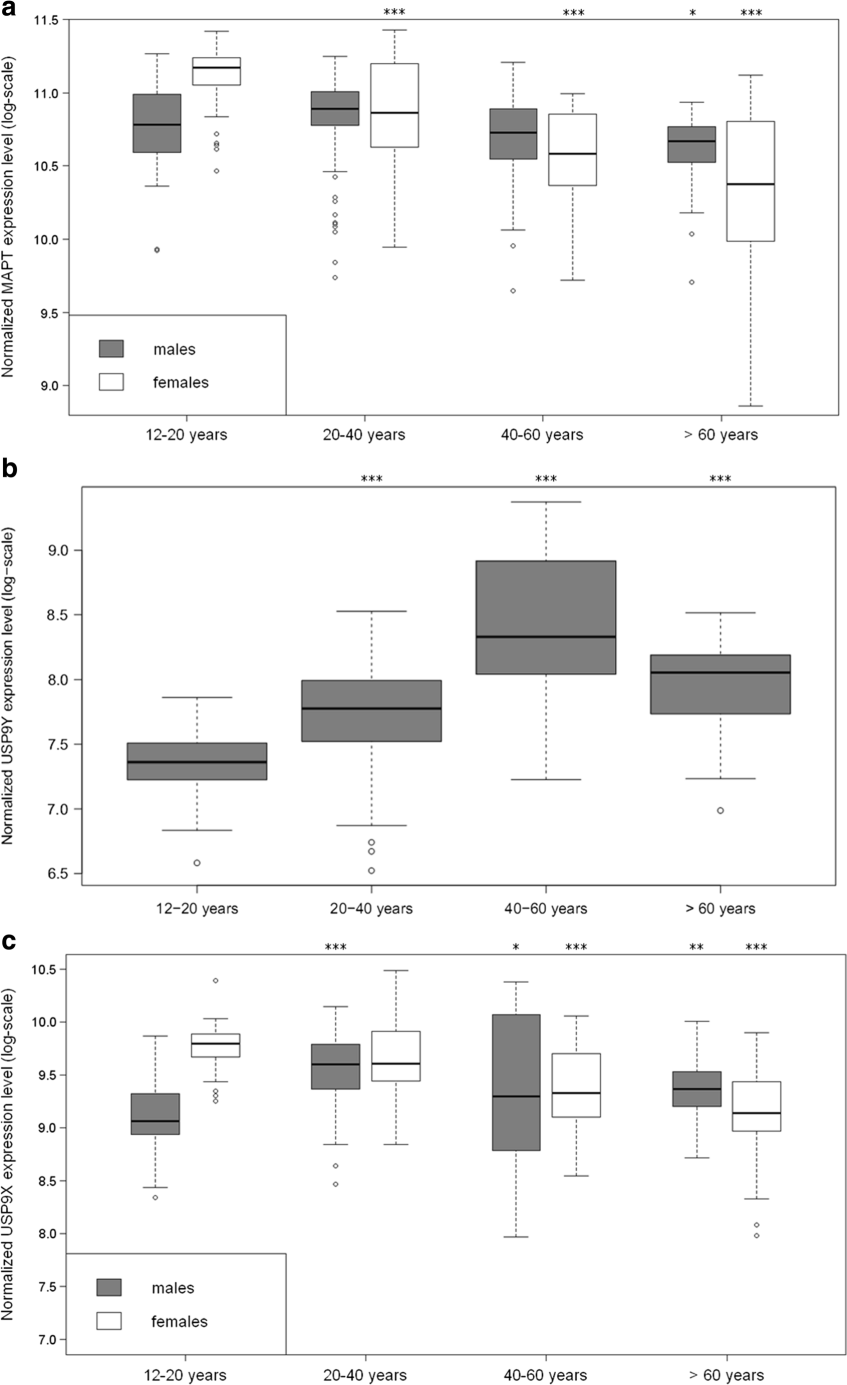
Boxplot for the normalized gene expression levels of **a** *MAPT*, **b** *USP9Y*, and **c** *USP9X* across different age groups in males and females in the Human Brain Transcriptome dataset (covering only individuals unaffected by AD). **a** After 20 years of age, median *MAPT* levels show a general trend of continuous decline with age but decrease faster in females as compared to males (two-sided Welch test *p* value significance relative to the 12–20 years age group in the same gender: *p < 0.05, **p < 0.01, ***p < 0.001). Comparing the differences between males and females for each age group, females have significantly higher *MAPT* levels in the 12–20 years group (*p* = 1.27E−08), whereas no significant difference is found in the 20–40 years group, and female *MAPT* levels are significantly lower in the 40–60 years group (*p* = 0.024) and the >60 years group (*p* = 0.0012). **b** Up to the age group of 40 to 60 years, *USP9Y* levels tend to increase with higher age (*p* value significance for males relative to 12–20 years age group: *p < 0.05, **p < 0.01, *** *p* < 0.001). **c** *USP9X* levels show no significant differences in males and females after 20 years of age, i.e., during adulthood the absence of *USP9Y* expression in females is not compensated by higher *USP9X* expression in females as compared to males (shown are only the significant *p* values relative to the 12–20 years age group in the same gender: *p < 0.05, **p < 0.01, ***p < 0.001)

*MAPT* and *USP9Y* expression levels were also compared across different adult age groups using brain transcriptomics data from individuals unaffected by AD from the Human Brain Transcriptome Project (HBT) project [14] (see the “Methods” section). In general, we observed a significant decrease in the levels of *MAPT* in the brain during healthy adult aging, matching with the results reported in an independent study [32]. Apart from this overall trend, we noted that the age-dependent decline in *MAPT* levels was gender-specific, with a faster decline in females as compared to males (see Fig. 2a). However, these gender differences in *MAPT* expression alone cannot explain the various gender differences in AD pathoetiology. Indeed, diverse types of gender differences have been observed across different *MAPT*-related diseases, e.g., a higher male preponderance for frontotemporal dementia [33] as opposed to an opposite risk profile in AD. While this indicates that *MAPT*-mediated gender intrinsic pathoetiology may be modulated also by other factors, it is perfectly in line with the role of *MAPT* in tauopathies.

Given the associations between *USP9* and *MAPT* described above, we also compared the genes in terms of the gender differences in their expression across different age groups. As shown in Fig. 2b, median *USP9Y* levels in males increase across the first three age groups (12 to 20, 20 to 40, and 40 to 60 years) with a slight relative decrease afterward. These changes in *USP9Y* were not compensated by corresponding increases in *USP9X* expression in females (see Fig. 2c).

Comparing Fig. 2a, b, the increased *USP9Y* expression in higher age groups in males coincides with a slower age-dependent decline of *MAPT* in males as compared to females, in line with the observed expression level correlations between *USP9Y* and *MAPT*. Since correlations do not necessarily imply a causal regulatory relationship and do not enable a distinction between cause and effect, we investigated a possible functional role of *USP9Y* as a positive regulator of *MAPT* by conducting *USP9* knockdown experiments in a zebrafish model and in the human DU145 cell culture model (see following sections).

### Tau Expression Is Decreased After USP9 Knockdown in Zebrafish

Among potential model organisms for the study of *MAPT* regulation, the zebrafish (*Danio rerio*) is of particular interest, since it has two *MAPT* paralogs, *mapta* and *maptb*, which reflect two major groups of MAPT isoforms in humans. While *maptb* is predominantly expressed as an isoform with three tubulin-binding repeats (3R-tau), *mapta* gives rise to isoforms with four to six repeats (4-6R-tau) [34, 35], which significantly increases its affinity for microtubules. Since 3R-tau and 4R-tau are the two main types of isoforms in humans, the two zebrafish paralogs may therefore provide a means to study how other regulatory genes influence the activity of the two isoforms.

**Fig. 3:**
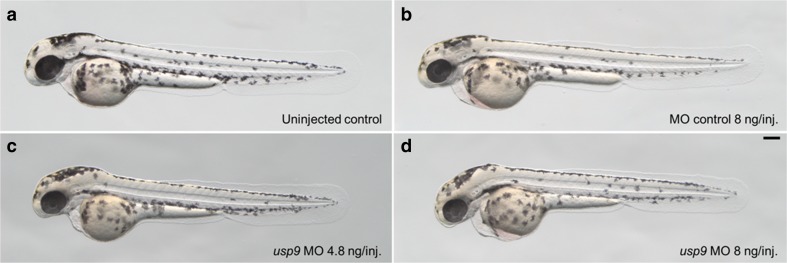
Representative pictures of *usp9* morphants and controls. Two-day-old *usp9* e2i2 morpholino injected larvae (**c** and **d**) displayed no phenotypic differences when compared to their corresponding controls (**a** and **b**; scale bar = 100 um)

The zebrafish is a well-established vertebrate model for the study of gene function and human pathologies [36, 37] and its genome displays a high degree of homology to the human genome [38]. In this study, we used zebrafish embryos as a model organism to investigate the possible regulatory relation between *USP9* and *MAPT*, by studying alterations in the expression of *mapta* and *maptb* in response to the knockdown of the zebrafish *USP9* ortholog (*usp9*). The knockdown was achieved using *usp9* antisense morpholino oligonucleotides (MO) blocking exon2-intron2 (e2i2) splicing. Gene expression changes were assessed via qRT-PCR (see the “Methods” section for details on the experiment and analysis). A microscopic analysis comparing uninjected control, morpholino control, and *usp9* MO-injected zebrafish showed no apparent toxic or developmental effect for the knockdown (see Fig. 3).

**Fig. 4:**
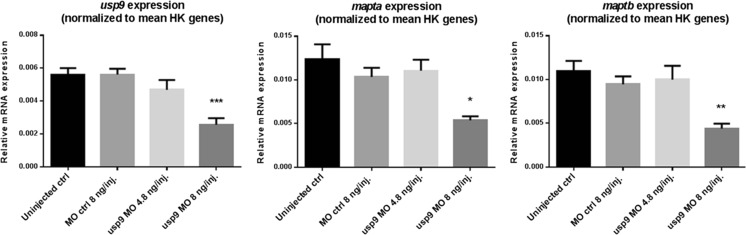
Boxplots for the normalized expression of the target genes *usp9* and *mapta* and *maptb*, assessed after *usp9* (e2i2) morpholino knockdown in 2 days postfertilization zebrafish embryos (*p < 0.05, **p < 0.01, ***p < 0.001)

As shown in Fig. 4 at 2 days postfertilization, gene expression in *usp9* morphants displays concentration-dependent decreases in both *mapta* and *maptb*, with a significant reduction at a MO concentration of 8 ng/injection (*p* < 0.05 for *mapta* and *p* < 0.01 for *maptb*). Overall, the results match with the high positive correlations between *USP9Y* and *MAPT* in the human *postmortem* transcriptomics datasets, further corroborating a possible regulatory relationship at the gene expression level.

### Transcriptome-Wide Analysis of USP9X/Y Knockdown Effects

#### Gene-Level Expression Analysis of USP9 Knockdown Effects in DU145 Cells

To assess the biomolecular effects of knocking down *USP9* on a transcriptome-wide scale, the human DU145 prostate cancer cell culture model was chosen, after confirming that *USP9X*, *USP9Y*, and *MAPT* are robustly expressed in this model. As a tumor-derived and non-neuronal cell line, DU145 can only provide an approximate model for the regulatory network around *USP9* in primary neuronal cells but has the advantage that stable expression of all genes of interest could be confirmed (see discussion of cell line choice and limitations in the “Methods” section).

ShRNA constructs were designed for the knockdown of *USP9X* and *USP9X/Y* (i.e., targeting *USP9X* and *USP9Y* jointly) to infer potential *USP9Y*-specific gene alterations indirectly by comparing both knockdowns. As discussed in the following paragraphs, no significant differences in the alteration patterns between the two knockdowns could be detected, prompting us to combine the knockdown data for the subsequent pathway analyses in the section “Pathway and network analysis of USP9 knockdown effects on gene expression in DU145 cells”. Multiple shRNA constructs were tested for knockdown efficiency and validated functionality via gene expression analysis in whole-cell populations. Only the best construct for each target was used for further experiments. Limitations regarding potentially undetected off-target effects when using a single shRNA construct per target, as well as the design and selection of constructs, are covered in the “Methods” section. As readout, microarray expression profiling using the Affymetrix GeneChip Human Gene 2.0 ST platform was performed with triplicate samples for each of the three considered conditions (*USP9X/Y* knockdown, *USP9X* knockdown, scrambled RNA control). This dataset has been made publicly available in the Gene Expression Omnibus (GEO) database under series GSE79376. Additionally, for target genes of interest discussed below, a qRT-PCR validation was performed (see the “Methods” section).

After preprocessing and normalization (see the “Methods” section), expression alterations were investigated first in the main genes of interest (*USP9X/Y* and *MAPT*), filtering the genetic probes mapping to these genes to retain only those with an average expression greater than the average expression across all probes on the chip. As expected, in the *USP9X/Y* knockdown, a marked underexpression was observed in comparison to the control samples for all 16 genetic probes mapping to the *USP9X* gene and fulfilling the average expression criterion (see detailed statistics in Suppl. Table S1). For *USP9Y*, 14 out of 15 genetic probes passing the average expression filter also showed a strong reduction in expression levels (see Suppl. Table S1; the only probe with a positive log-fold change displayed a higher than average standard deviation and is therefore likely an artifact of noise). These expression level decreases in the *USP9X/Y* knockdown were confirmed in the qRT-PCR validation (*p* = 4.6E−10 for *USP9X* and *p* = 4.77E−07 for *USP9Y*). For the *USP9X*-specific knockdown, an underexpression trend was also confirmed (see Suppl. Table S1).

**Fig. 5:**
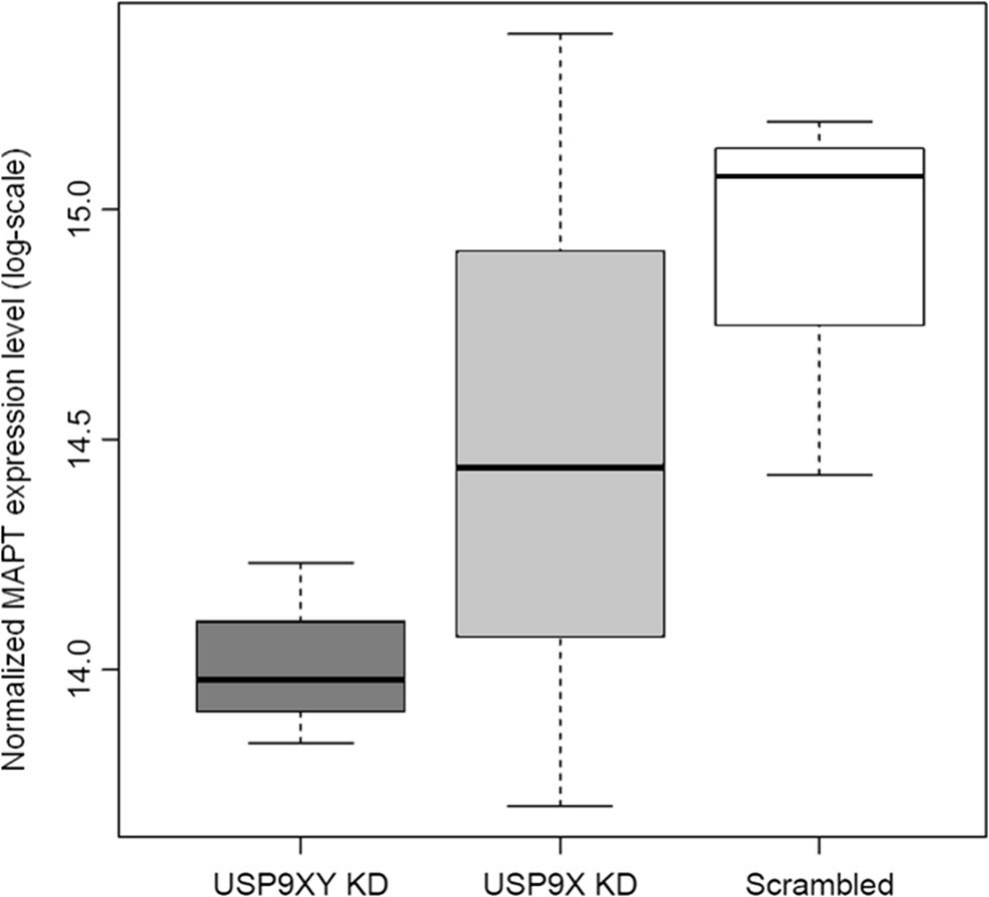
Normalized *MAPT* expression levels on log-scale in the *USP9XY* and *USP9X* knockdowns (KD) and the scrambled control (scrambled) for the *MAPT* genetic probe with the highest average expression in the DU145 microarray dataset. Decreased median *MAPT* expression levels are observed for both knockdowns (*p* = 0.017 for the *USP9XY* knockdown and *p* = 0.44 for the *USP9X* knockdown)

Interestingly, gene expression levels for *MAPT* were consistently decreased for the *USP9X/Y* knockdown for all of eight filtered *MAPT* probes and in the qRT-PCR validation, as well as for all seven probes passing the abovementioned average expression filter for the *USP9X* knockdown (see Suppl. Table S1). These results are in line with the observed underexpression *of mapta* and *maptb* in the zebrafish *usp9* knockdown (see Figs. 4 and 5). Moreover, the *MAPT* probe expression levels displayed positive average correlations with those for the *USP9X* probes (avg. Pearson correlation across all pairs of filtered probes: *r* = 0.479, avg. *p* = 0.096) and *USP9Y* probes (avg. *r* = 0.472, avg. *p* = 0.082). Similar correlations were also observed in the AD brain transcriptomics datasets by Liang et al. [20] (*r* = 0.483, *p* = 2.3E−07) and Blalock et al. [21] (*r* = 0.425, *p* = 0.169).

**Fig. 6:**
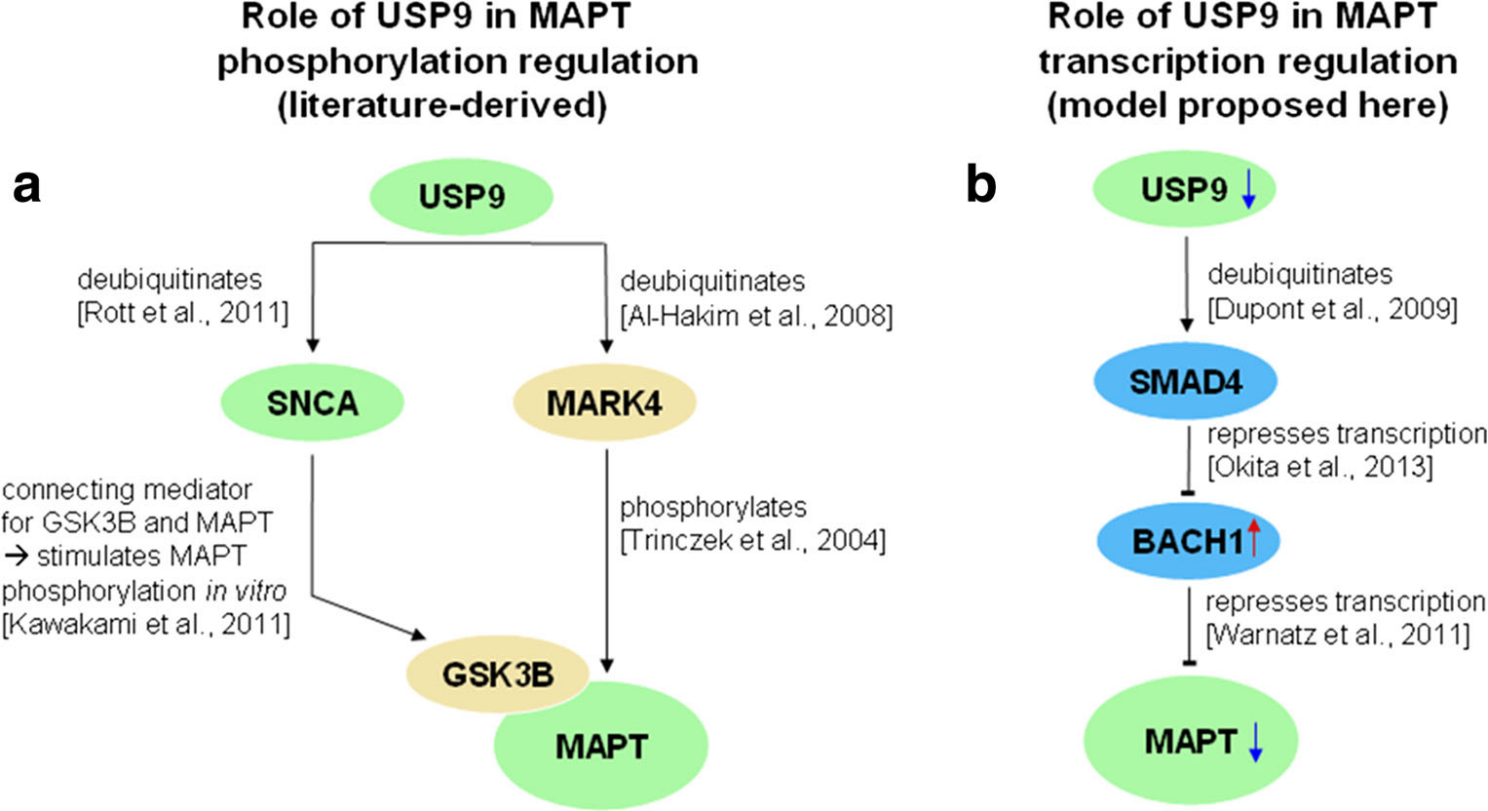
**a** Role of USP9 in the regulation of MAPT phosphorylation using relations derived from the literature (see labels of the directed edges). **b** Proposed model for the role of USP9 in the regulation of *MAPT* gene expression, derived from the upstream analysis of the DU145 transcriptomics data. In both **a** and **b**, genes/proteins are represented by *ellipses* and colored *blue* for transcription factors, beige for kinases, and *green* for all other proteins. Small *blue arrows pointing downward* represent genes with decreased mRNA levels after *USP9X/Y* knockdowns in DU145 cells, and *red arrows pointing upward* highlight increased mRNA levels observed after the knockdowns

To identify possible molecular mechanisms behind the underexpression of *MAPT* in the *USP9X/Y* and *USP9X* knockdowns, we conducted an upstream network analysis, investigating transcription factors (TFs) known to be involved in the regulation of *MAPT* transcription for gene expression alterations in our dataset, using literature-curated TF-target relationships from the Biobase Proteome™ database (see http://www.biobase-international.com/proteome-2), the ResNet® Mammalian database 11 from Elsevier (see http://www.ariadnegenomics.com), and GeneCards [18]. *MAPT* transcriptional regulators were filtered according to the consistency of their expression alterations and the decrease of *MAPT* levels observed in the DU145 *USP9X/Y* knockdown gene expression data with their reported regulatory effect on *MAPT*. One known *MAPT* transcription repressor, *BACH1* [39], was found to have increased expression levels in the *USP9X/Y* knockdown for five out of seven filtered genetic probes and for seven out of eight filtered probes for the *USP9X* knockdown (see Suppl. Table S1). By investigating *BACH1* upstream regulators using regulatory interactions from the ResNet Mammalian database, a mechanistic link between *BACH1* and *USP9* was found. Since USP9 is known to deubiquitinate SMAD4 [16], loss of USP9 orthologs disables SMAD4-dependent responses in several model systems [16], and *SMAD4* knockdown has been shown to constitutively activate *BACH1* expression [40], and the *USP9X/Y* knockdown effect on *BACH1* expression and its downstream target *MAPT* can be explained by an inhibition of the common monoubiquitinated SMAD4 (see also our proposed mechanistic model for the involvement of *USP9* in *MAPT* expression regulation in Fig. 6b). Specifically, SMAD4 is monoubiquitinated by the E3 Monoubiquitin Ligase TRIM33 (Ectodermin/Tif1γ), inhibiting SMAD4 responses, an action which is opposed by the deubiquitinase USP9 [16].

Overall, in spite of a higher variance across replicate samples in the *USP9X* knockdown, a highly significant Pearson correlation between the log-fold expression changes for the *USP9X/Y* and the *USP9X* knockdown was observed both across the entire DU145 transcriptomics dataset (*r* = 0.56, *p* < 2.2E−16) and in particular when considering only the top 1000 most significant shared genetic probes (*r* = 0.965, *p* < 2.2E−16), which are expected to be less strongly affected by random variation than the entire set of transcripts. Furthermore, no significant difference in the alterations between the significantly differentially expressed genes in the *USP9X/Y* knockdown and the *USP9X* knockdown could be detected. This observation may result from a shared functional profile of *USP9X* and *USP9Y*, or alternatively, from a lack of statistical power to detect smaller differences in downstream gene expression changes or an insufficient specificity of the knockdowns. In the *USP9X* knockdown, a small but not statistically significant decrease in *USP9Y* expression was observed in the qRT-PCR measurements (*p* = 0.759), which may indicate a weak off-target effect of this knockdown on *USP9Y*. Regarding the possibility of a lack of statistical power to reliably detect small gene expression differences between the knockdowns, this cannot be excluded, but the high correlation of fold changes observed across the top 1000 shared genetic probes, which include a majority of probes with small effect sizes in their alterations in the individual differential expression analyses, does not point to any significant shifts in expression pattern, and no clear outliers were observed that would indicate a biologically meaningful difference for specific genes between the knockdowns. Alterations for genetic probes ranked lower than the top 1000 have too small effect sizes or too high variation to be distinguished from random variation (these probes all have adjusted *p* values for differential expression of at least 0.45). In summary, we could not detect robust gene expression differences between the knockdowns or indications of such differences that would warrant a qPCR validation and could be used for biological data interpretation. Although we cannot exclude the existence of functional differences between the X- and Y-forms of *USP9* due to the limitations of the omics profiling and knockdown approach, our results rather support the hypothesis that *USP9*-related gender differences result solely from differences in total *USP9* expression levels (i.e., the sum of *USP9X* and *USP9Y* expression levels). Given the highly correlated alteration patterns at the level of single genes in the two knockdowns, the lack of detectable statistically significant differences between the knockdowns, and the high protein sequence similarity between USP9X and USP9Y of 93%, which also suggests a high structural and functional similarity, we have therefore decided to combine the *USP9X/Y* and *USP9Y* knockdown groups for the subsequent analyses to increase the statistical power for the investigation of pathway and network alterations.

#### Pathway and Network Analysis of USP9 Knockdown Effects on Gene Expression in DU145 Cells

To identify cellular pathways with jointly altered activity in the *USP9XY* and *USP9X* knockdowns, a pathway enrichment analysis was performed using the GeneGO software (see Methods). Table 2 reports the top ten cellular pathways with an overrepresentation of differentially expressed genes in the knockdowns. These altered pathways mainly include processes related to cytoskeleton remodeling, cell adhesion, and stress signaling (ordered by significance).

**Table 2:** Top ten cellular pathways enriched in differentially expressed genes in the *USP9XY* and *USP9X* knockdown samples as compared to the controls, sorted by *p* value significance (column headers are defined as follows: Total = total number of biomolecules in the pathway; FDR = false-discovery rate; In Data = total number of pathway members among the differentially expressed genes in the *USP9XY* knockdown samples)

No.	GeneGO top-ranked pathways	Total	*p* value	FDR	In Data
1	Cytoskeleton remodeling	102	3.49E−16	3.09E−13	84
2	TGF, WNT and cytoskeletal remodeling	111	9.24E−16	4.10E−13	89
3	Role of PKR in stress-induced antiviral cell response	57	3.81E−11	1.13E−08	50
4	Positive regulation of STK3/4 (Hippo) pathway and negative regulation of YAP/TAZ function	70	7.25E−11	1.61E−08	58
5	Chemokines and adhesion	100	3.23E−10	5.74E−08	75
6	EGFR signaling pathway	71	9.75E−09	1.44E−06	56
7	Epigenetic regulation of gene expression	57	2.51E−08	2.87E−06	47
8	SLE genetic marker-specific pathways in antigen-presenting cells (APC)	84	2.59E−08	2.87E−06	63
9	TNFR1 signaling pathway	43	2.98E−08	2.93E−06	38
10	Regulation of epithelial-to-mesenchymal transition (EMT)	64	4.13E−08	3.66E−06	51

A significance threshold for individual genes of *p* < 0.05 was used, and only pathways with a minimum of ten mappable genes were considered

Studying the molecular network regions associated with cytoskeleton remodeling in more detail revealed pronounced expression changes in several tubulins and microtubule-associated genes (e.g., *TUBA1B*, *TUBA3C*, *TUBA3E*, *TUBA4B*, *TUBB2A*, *TUBB4A*, *TUBB8*, *MAP2*, and *MAP4*). Overall, tubulins tend to have reduced expression in the knockdowns with few exceptions (e.g., *TUBA8* and *TUBG2*). Supplementary Fig. S1 shows a corresponding subnetwork from the pathway “Regulation of cytoskeleton proteins in oligodendrocyte differentiation and myelination” (adjusted *p* value: 2.28E−05). More specifically, the figure highlights that tubulins involved in binding interactions with microtubule-associated protein tau (MAPT) are underexpressed, matching with the decreased expression observed for *MAPT* itself (see above). These results suggest that the underexpression of *MAPT* in the *USP9XY*/*USP9X* knockdowns is part of a broader response affecting a subnetwork involving *MAPT* and tubulins within cytoskeleton regulation-associated processes. The increased expression of the transcription factor *BACH1*, which represses *MAPT* expression, may partly explain these changes, since multiple tubulins are predicted as further target genes repressed or activated by *BACH1* according to the SABioscience DECODE database (www.sabiosciences.com), including *TUBA1A*, *TUBA1C*, *TUBA4A*, *TUBA4B*, *TUBA8*, *TUBB*, *TUBB2A*, *TUBB2C*, *TUBB3*, *TUBB6*, *TUBD1*, *TUBG2*, and *TUBGCP2*. The DECODE database contains binding site predictions derived from SABioscience’s proprietary Text Mining Application and manual genome annotations from the UCSC Genome Browser [41] (the database was queried via the SABioscience “Champion ChiP Transcription Factor Search Portal” at www.sabiosciences.com, searching for the transcription factor *BACH1* and setting the species to “Human”). Interestingly, USP9X has previously also been found to associate with microtubules in neuronal processes and to interact in vivo with doublecortin (DCX), a microtubule-associated protein involved in neuronal migration [42].

#### Network Model of USP9 Knockdown Effects on MAPT Phosphorylation and Expression

In Fig. 6, we combine information from the literature on the involvement of USP9 in MAPT phosphorylation regulation (Fig. 6a) and our findings from the upstream network analysis in a mechanistic model for *USP9*-mediated regulation of *MAPT* gene expression (Fig. 6b). As outlined in Fig. 6a, previous evidence from the literature shows that USP9 can influence the phosphorylation of MAPT both by deubiquitinating the kinase MARK4 [26], which directly phosphorylates MAPT, and by deubiquitinating SNCA [30], which mediates the connection between glycogen synthase kinase 3β (GSK3B) and its phosphorylation target MAPT [31]. *MAPT* expression can be altered by USP9-mediated deubiquitination of the transcription factor SMAD4 [16], which represses *BACH1* transcription. *BACH1*, in turn, encodes a transcriptional repressor for *MAPT* [39] (see Fig. 6b).

Additional functional relationships between USP9 and the cytoskeleton are suggested by the interactomes of MARK4 and BACH1. Apart from MAPT, MARK4 also phosphorylates further tau family microtubule-associated proteins (MAPs) [27]. The transcriptional *MAPT* repressor BACH1 has several additional tubulin targets, explaining why tubulins are underexpressed in the *USP9X/Y* knockdown (see Suppl. Fig. S1).

Interestingly, MARK phosphorylation of tau family MAPs has been shown to trigger microtubule disruption [43], and inhibitors of tau and MAP phosphorylation have previously been proposed as drug targets for Alzheimer’s disease (AD). For example, GSK3B inhibition via small molecule ligands has been suggested as a possible therapeutic intervention strategy to counteract MAPT hyperphosphorylation in AD [44], and heat shock protein 70 (HSP70), which suppresses alpha-synuclein (SNCA)-induced MAPT phosphorylation via GSK3B through direct binding to SNCA, has also been proposed as a therapeutic target [31]. In a similar fashion, modulation of *USP9X/Y* activity could be of interest as a new intervention strategy to alter both the phosphorylation of MAPT (via MARK4 or SNCA/GSK3B, as outlined in Fig. 6a) and *MAPT* expression levels (via SMAD4 and BACH1, see Fig. 6b).

## Conclusions

The human brain transcriptome displays several significant gender differences with large effect size, which may influence the risk for brain disorders and the severity of their phenotypic manifestations. In particular, in this study, we observe gender-specific differences in the age-dependent decrease of brain gene expression levels for the microtubule-associated protein tau (MAPT), a protein playing a central role in neurodegenerative diseases referred to as tauopathies. In a joint analysis of multiple transcriptomics datasets from independent studies, we identify *USP9* as a candidate MAPT regulator behind these gender differences. Previous studies have shown that USP9 can influence the phosphorylation of MAPT via deubiquitination of the MAPT-phosphorylating kinase MARK4 and indirectly via deubiquitination of SNCA, which stimulates MAPT phosphorylation by mediating its connection with the kinase GSK3B. At the gene transcription level, *USP9* knockdown experiments in zebrafish and the DU145 human cell culture result in reduced *MAPT* levels, confirming the positive correlation between *USP9* and *MAPT* observed in human brain transcriptomics datasets.

A mechanistic explanation for this correlation is provided by the upstream network analysis of the *USP9* knockdown transcriptome, exploiting existing molecular interaction data and identifying a regulatory relation between *USP9* and *MAPT* via the USP9 deubiquitination target SMAD4 and the *MAPT* transcription repressor BACH1, whose transcription is in turn repressed by SMAD4. This potential pathway merits further validation and investigation with regard to a putative relevance for AD molecular pathology and intervention strategies. Since the transcription factor BACH1 is predicted to target multiple tubulins and the kinase MARK4 is involved in the phosphorylation of multiple microtubule-associated proteins (MAPs), the alterations in these regulators also provide a parsimonious explanation for the main changes observed in the pathway and network analysis of the USP9 knockdown effects. Overall, the pathway/network analysis shows that cellular processes related to cytoskeleton remodeling, as well as tubulins and MAPs in the interaction network around MAPT, are most significantly affected.

In summary, in the context of prior knowledge from the literature, the presented transcriptomics data supports the existence of mechanistic links between USP9 and MAPT and suggests that USP9 could be of biomedical interest as a regulator modulating both the expression and phosphorylation of MAPT. For tauopathies like Alzheimer’s disease, involving aggregations of paired helical filaments of MAPT into neurofibrillary tangles, USP9 may therefore warrant further study as a target for the development of new intervention strategies.

## Materials and Methods

### Gene Expression Data Processing and Analyses

All raw microarray datasets from Affymetrix platforms were preprocessed and normalized using the GC-RMA approach [45]. For the dataset from the study by Zhang et al., derived from Rosetta/Merck Human 44 k 1.1 chips and not suitable for the GC-RMA procedure, the preprocessed data according to the procedures in the original publication was used [19]. Differential gene expression was scored and analyzed using the empirical Bayes moderated t-statistic [46], and the resulting *p* value significance scores were adjusted for multiple hypothesis testing following the approach by Benjamini and Hochberg [47].

To account for differences in brain regions covered across the microarray datasets used, we focused on genes that displayed consistent, multiregional expression changes in the HBT dataset [14] (NCBI GEO dataset series GSE25219) and the late-onset AD case/control dataset [19] (NCBI GEO dataset series GSE44772), i.e., genes with consistent signs for the logarithmic fold changes and with overall significant *p* values after adjustment for multiple hypothesis testing (adjusted *p* value <0.05). While the GSE25219 dataset covers 16 brain regions (see the section “USP9Y displays significant gender-linked expression in the adult brain and diminished expression in Alzheimer’s disease”), the GSE44772 dataset covers three regions (the prefrontal cortex, visual cortex, and cerebellum, which represent higher-level groupings of brain regions that overlap with the 16 brain regions for the GSE25219 dataset). The reason for focusing on genes with consistent expression alterations across multiple brain regions is that additional evidence for corresponding multiregional alteration patterns can be collected across datasets from other studies, which cover the same or further brain regions. For the gene of interest derived from the analyses on the GSE25219 and GSE44772 dataset, *USP9Y*, which showed both consistent multiregional gender-linked expression (GSE25219 dataset) and multiregional gender-specific expression alterations in AD (GSE44772 dataset), two further microarray datasets were studied to investigate whether the significantly reduced expression in male AD patients as compared to unaffected male subjects observed on the GSE44772 dataset across the prefrontal cortex, visual cortex, and cerebellum, could also be found in the brain regions covered by other AD case/control datasets. Specifically, significant multiregional expression changes for *USP9Y* were confirmed in the NCBI GEO dataset GSE5281 [20] (covering the brain regions primary visual cortex, entorhinal cortex, hippocampus, medial temporal gyrus, posterior cingulated, and superior frontal gyrus), and a matching qualitative change was observed in hippocampal samples of the NCBI GEO dataset GSE28146 [21] (see the section “USP9Y displays significant gender-linked expression in the adult brain and diminished expression in Alzheimer’s disease”).

A limitation of these investigations of consistent gene expression alterations across multiple brain regions is that the quantitative statistical results for specific brain regions on a single dataset cannot be directly integrated with the statistics obtained from independent datasets covering distinct brain regions to increase the statistical power. However, by using datasets with large sample sizes and coverage of multiple brain regions to identify genes with multiregional consistent expression changes, a qualitative confirmation of multiregional expression alterations is possible on independent datasets, which may partly cover distinct brain regions (comparing the statistics on the separate datasets, instead of integrating them into a single statistic).

To exclude age-related biases in the significant findings for the analyses of microarray datasets from the AD case/control studies, the significance of gene expression alterations between patients and controls was confirmed after adjustment for age using a surrogate variable analysis (R software package SVA [48]). As a separate investigation related to aging-associated changes, a transcriptome-wide analysis and comparison of brain gene expression alterations during adult aging and in the neurodegenerative disorders Alzheimer’s and Parkinson’s disease has been presented previously [49].

To conduct cellular pathway analyses, the differential expression analysis results for the combined knockdown samples as compared to the control samples were used as input for the GeneGO pathway analysis software [50], filtering out genetic probes with a *p* value above 0.05. Pathways with an overrepresentation of differentially expressed genes were ranked by increasing *p* value. For the upstream network analysis, literature-curated TF-target relationships from the Biobase Proteome™ database (see http://www.biobase-international.com/proteome-2) and GeneCards [18] were used, and the consistency of potentially relevant TF-target pairs with the alteration patterns observed in the transcriptomics data was checked.

For qRT-PCR measurement analyses, the data was normalized against the mean of multiple reference genes according to the method by Hellemans et al. [51] (see details on the selected reference genes and the experimental design in the “Method” sections on the zebrafish and cell culture experiments). The Welch’s *t* test was then applied to assess the significance of differential expression (changes with a significance of *p* < 0.05 are highlighted by star symbols in the corresponding boxplots, see Fig. 4).

### Zebrafish Experiments

#### Knockdown Experiments

The *usp9* antisense morpholino (MO) was designed and synthesized to target the splice donor site of exon 2 (5′-TGAAAATGGTGCTCTGACCTGGTTC-3′) and to interfere with normal pre-mRNA splicing of the zebrafish *usp9* gene (ENSDART00000135384). To analyze gene expression changes after *usp9* antisense morpholino MO knockdown in zebrafish for the target genes *usp9*, and the zebrafish MAPT paralogs *mapta* and *maptb*, titrated microinjections were performed with 4.8 and 8 ng/injection of an e2i2 splice blocking *usp9* MO in one to two cell-stage zebrafish embryos. Control MO (randomized 25 N oligomer, 8 ng/injection) and wild-type embryos (non-injected controls) were processed in parallel. All MOs were designed and synthesized by Gene Tools. Embryos were maintained in standard conditions (28 °C) in embryo medium (0.3× Danieau’s). Two days after microinjection, 20 dechorionated embryos per condition were processed for RNA extraction and reverse transcription. For each target gene to be assessed via qRT-PCR gene expression measurements (LightCycler®480, Roche), three different pairs of primers were initially tested for specificity, and then the most specific pair was selected for further experiments (see Suppl. Table S2). Three zebrafish housekeeping (HK) genes were processed in parallel (*β-actin1*, *elongation factor 1*, and *60S ribosomal protein L13*). The qRT-PCR was performed with five biological replicates per condition, and each biological replicate was covered by four technical replicates.

### Cell Culture Experiments

In order to study *USP9X/Y* and its gene regulatory network in a cell culture model, relevant human cell lines were first compared in terms of the stability of *USP9X*, *USP9Y*, and *MAPT* and expression using information from the literature and public gene expression data. The prostate carcinoma cell line DU145 was chosen, since the public transcriptomics data showed stable expression for the genes of interest (*USP9Y*, *USP9X*, *MAPT*), as opposed to the alternative neuroblastoma cell line from female origin SH-SY5Y (ATCC no. CRL-2266, undifferentiated) and the human embryonic kidney cell line HEK293 (ATCC no. CRL-1573, see Suppl. Fig. S2 and S3 and Suppl. Tables S3 and S4). Since DU145 cells are derived from a non-neuronal tumor tissue, the molecular regulatory network around *USP9* present in this cell line can only provide an approximation to a corresponding network in a genetically stable, neuronal cell population. Possible limitations include that yet unknown regulatory genes in this network and downstream effectors of *USP9* with tissue-specific expression in primary neuronal cells may not be expressed in DU145 cells, and that like in other tumor cell lines, the metabolism may be shifted toward proliferation and growth. We therefore consider the analysis of *USP9* in DU145 cells in combination with our findings in the zebrafish model, the analyses of human brain microarray data, and information from the published literature, to avoid over-reliance on a single model and information source.

DU145 cells were obtained from the American tissue culture collection (ATCC no. HTB-81). Cells were cultured in Dulbecco’s modified eagle medium (Invitrogen no. 41966-029) containing high glucose (25 mM), l-glutamine (4 mM), and sodium pyruvate (1 mM). This medium was supplemented with 10% *v*/*v* heat-inactivated fetal bovine serum (Invitrogen no. 10500-064). Cells were grown at 37 °C at 5% CO2 and saturated humidity.

Knockdown plasmids were designed to either target *USP9X* and *USP9Y* jointly or *USP9X* specifically. Due to the high sequence homology between *USP9X* and *USP9Y*, the production of potentially unspecific *USP9Y* shRNA plasmids was omitted. Hairpin sequences (Suppl. Tab. S5) and U6 promoters were subcloned into FastBac plasmids (PMID 15771966). The list of all primers tested for the knockdown experiments is provided in Suppl. Tab. S3, and the agarose gel electrophoresis results are shown in Fig. S3. While multiple shRNA constructs per target gene were designed and tested for knockdown efficiency via gene expression analysis in whole-cell populations, for each target only, the construct with the best knockdown efficiency was used for subsequent profiling. As the efficiency of gene silencing may vary for different shRNA constructs and off-target effects can occur in some cases, limitations may arise in the interpretation of data derived from the use of a single construct. However, since the highly significant inhibition of the target genes observed in both microarray and qRT-PCR expression measurements (see the section “Gene-level expression analysis of USP9 knockdown effects in DU145 cells”) was not matched or approximated by expression changes observed in any other gene in the differential expression ranking derived from the microarray data, at least a strong off-target alteration can be excluded, as well as a scenario in which a larger amount of the target transcripts escapes the inhibition to recover the normal function. Transfection into DU145 was done via Lipofectamine 2000 (Invitrogen no. 11668019) and transfected cells were incubated for 48 h. Prior to RNA extraction, the perturbed cells were enriched via fluorescence-activated cell sorting. RNA was extracted using the Qiagen RNeasy Mini Kit (Qiagen no. 74106) and treated with DNaseI (Qiagen no. 79254). Reverse transcription was done as described previously (PMID 26738520). For microarray expression profiling, RNA extracts were prepared using the GeneChip WT PLUS Reagent Kit (Affymetrix, Manual P/N 703174 Rev. 2 and UserGuide GeneChip Expression Wash, Stain and Scan for Cartridge Arrays P/N 702731 Rev. 4). RNA quality and integrity was checked using a NanoDrop ND-100 spectrophotometer (Thermo Scientific) and the 2100 Agilent Bioanalyzer (Agilent), respectively. The purified, sense-strand cDNA was fragmented by uracil-DNA glycosylase (UDG) and apurinic/apyrimidinic endonuclease 1 (APE 1) at the unnatural dUTP residues. The fragmented cDNA was labeled by terminal deoxynucleotidyl transferase (TdT) using the Affymetrix proprietary DNA Labeling Reagent that is covalently linked to biotin. Single-stranded cDNA (5.5 μg) was used for fragmentation and labeling, and the GeneChip Hybridization, Wash and Stain Kit was used to hybridize and wash the cartridges. Control Oligonucleotide B2 and 20X Eukaryotic Hybridization Controls were added to the hybridization cocktail containing the labeled sample and injected into the cartridge. The incubation lasted 16 h at 45 °C with a rotation at 60 rpm. Then, the Fluidics Station 450/250 was used to wash and stain the Affymetrix GeneChip Human Gene 2.0 ST probe arrays. The probe arrays were scanned after completion of the wash protocols using the Affymetrix GeneChip Scanner 3000.

Data was processed as described in the “Methods” section on gene expression data processing and analysis. Both the raw and processed data have been deposited in the Gene Expression Omnibus (GEO) database under the series accession number GSE79376.

For validation of microarray-derived gene expression data in USP9 knockdown conditions, shRNA perturbations in DU145, cell sorting, RNA extraction, and reverse transcription were redone as described above. Real-Time PCR expression measurements for the target genes of interest and six reference genes were performed using a Fluidigm 48.48 integrated fluidic circuit array. The six reference genes consisted of three genes commonly used as reference for human cell culture RNA expression measurements (*PPIA*, *GAPDH*, and *PDHB*) and three genes displaying the lowest variation in the processed Affymetrix GeneChip microarray data for the DU145 cells (*EIF4G2M*, *HSPA8*, and *UBAP2L*). The assay IDs and catalog numbers for the TaqMan® gene expression assays used for the target and reference genes are provided in Suppl. Table S6. For each condition (*USP9X/Y* knockdown, *USP9X* knockdown, and three scrambled RNA control samples) measurements were obtained for five biological replicates.

To preprocess and analyze the raw data from the Fluidigm platform, an optimal subset of two reference genes (*PPIA* and *HSPA8*) was determined using the method by Vandesompele et al. [52] as implemented in the R software package SLqPCR (http://www.bioconductor.org). After determining the median across the measurements for the selected reference genes, a ΔCt calculation was performed as described in Yuan et al. [53] and the empirical Bayes moderated t-statistic [46] was applied to compare *USP9X/Y* knockdown samples against controls.

## ELECTRONIC SUPPLEMENTARY MATERIAL

ESM 1 Supplementary Material (file: supplementary_material.pdf): The Supplementary Material contains tables of the differential expression statistics in the microarray transcriptomics data for DU145 cells, comparing *USP9X/Y* knockdown samples vs. controls, for the main genes discussed in the manuscript (Tab. S1), the zebrafish primer design (Tab. S2), the PCR program, primers and hairpin sequences for the DU145 cell culture experiments (Tab. S3, S4 and S5), and the assay IDs and catalog numbers for the TaqMan® gene expression assays (Tab. S6). Moreover, figures are provided for the molecular network analysis of the DU145 transcriptomics data (Fig. S1), the primer design scheme for the DU145 knockdown experiments (Fig. S2), and the agarose gel electrophoresis results for testing different primers for the cell culture experiments (Fig. S3) (PDF 573 kb)
