# Supplementary material for: Gender-Specific Expression of Ubiquitin-Specific Peptidase 9 Modulates Tau Expression and Phosphorylation: Possible Implications for Tauopathies
Source: Mol Neurobiol. 2016 Nov 23;54(10):7979–93. doi: 10.1007/s12035-016-0299-z (PMC5684262; doi:10.1007/s12035-016-0299-z)
Supplement: Supplementary file 1 — Supplementary Material (file: supplementary_material.pdf): The Supplementary Material contains tables of the differential expression statistics in the microarray transcriptomics data for DU145 cells, comparing USP9X/Y knockdown samples vs. controls, for the main genes discussed in the manuscript (Tab. S1), the zebrafish primer design (Tab. S2), the PCR program, primers and hairpin sequences for the DU145 cell culture experiments (Tab. S3, S4 and S5), and the assay IDs and catalog numbers for the TaqMan® gene expression assays (Tab. S6). Moreover, figures are provided for the molecular network analysis of the DU145 transcriptomics data (Fig. S1), the primer design scheme for the DU145 knockdown experiments (Fig. S2), and the agarose gel electrophoresis results for testing different primers for the cell culture experiments (Fig. S3) (PDF 573 kb) [file 12035_2016_299_MOESM1_ESM.pdf]

Supplementary Materials for "Gender-specific expression of ubiquitin-specific peptidase 9 modulates tau expression and phosphorylation: possible implications for tauopathies"

Sandra Köglsberger, Maria Lorena Cordero-Maldonado, Paul Antony, Julia Ilona Forster, Pierre Garcia, Manuel Buttini, Alexander Crawford, Enrico Glaab†

Luxembourg Centre for Systems Biomedicine (LCSB), University of Luxembourg, 7 avenue des Hauts Fourneaux, L-4362 Esch-sur-Alzette, Luxembourg

†Corresponding author: enrico.glaab@uni.lu, T: +352 466644 6186, Fax: +352 466644 6949

1.	Table S1	p. 2
2.	Table S2	p. 3
3.	Table S3	p. 4
4.	Table S4	p. 4
5.	Table S5	p. 5
6.	Table S6	p. 5
6.	Figure S1	p. 6
7.	Figure S2	p. 7
8.	Figure S3	p. 8

Knockdown target	Measured gene	Min. p-value	Max. p-value	Min. log. fold change	Max. log fold change
USP9XY	USP9X	1.72E-07	9.98E-03	-4.19	-1.22
	USP9Y	5.22E-05	3.95E-02	-2.23	-0.84
	MAPT	0.017	0.38	-1.24	-0.5
	BACH1	0.025	0.87	0.06	0.72
USP9X	USP9X	0.009	0.87	-1.42	-0.11
	MAPT	0.032	0.44	-1.26	-0.27
	BACH1	0.0054	0.30	0.48	0.84

Tab. S1 Differential expression statistics under different knockdown experiments (USP9XY and USP9X, both as compared to controls) and for the main genes discussed in the manuscript and measured in the transcriptomics data for DU145 cells. The statistics correspond to the maximum and minimum p-values and log transformed fold changes obtained with the empirical Bayes moderated t-statistic across the pre-filtered genetic probes for the assessed genes (the pre-filtering removed all probes with an average expression below the overall average expression in the transcriptome dataset).

a)

Primer pairs	Sequence (5' → 3')	Start	Stop	Tm	GC%	Self complementarity	Self 3' complementarity
*Forward 1	ACCAACCCCCACTGTAAGTT	532	551	59.97	50.00	4.00	3.00
*Reverse 1	AATCCACCAACCAACCCTTG	670	651	59.46	50.00	3.00	3.00
**Product length: 139 bp**							
Forward 2	AGCCTTCATCAGCCACAGTA	1207	1226	59.90	50.00	3.00	2.00
Reverse 2	CCTCATGTTTCCCAGCCTGA	1330	1311	60.85	55.00	5.00	3.00
**Product length: 124 bp**							
Forward 3	TGACGGAGAACGGGATGAAA	2195	2214	60.19	50.00	2.00	0.00
Reverse 3	AATCACAACCCTCCACAGGT	2337	2318	60.04	50.00	3.00	2.00
**Product length: 143 bp**							
mapt a (XM_001340530.4): *Danio rerio* microtubule-associated protein tau a mRNA, 2276 bp							
**Primer pairs**	**Sequence (5' → 3')**	**Start**	**Stop**	**Tm**	**GC%**	**Self complementarity**	**Self 3' complementarity**
*Forward 1	CGCACAGTATCCTCGTTTGG	68	87	60.12	55.00	4.0	0.00
*Reverse 1	CCAGAGAGGGAAGAAGCCAT	177	158	59.97	55.00	2.00	2.00
**Product length: 110 bp**							
Forward 2	ATGGCTTCTTCCCTCTCTGG	158	177	59.97	55.00	2.00	0.00
Reverse 2	CTTGGATTGAGCTGTGGGTG	277	258	59.98	55.00	4.00	0.00
**Product length: 120 bp**							
Forward 3	TCACAAACCAGGTGGAGGAA	1201	1220	59.97	50.00	5.00	0.00
Reverse 3	ACGGAACGTCAGTTTGTGTG	1345	1326	60.12	50.00	4.00	0.00
**Product length: 145 bp**							
mapt b (XM_002661150.3): *Danio rerio* microtubule-associated protein tau b mRNA, 1312 bp							
*Forward 1	GCTCCAAGGCCAACATTCAT	928	947	59.97	50.00	4.00	2.00
*Reverse 1	TGTGGCTCTCAATCCTCCTC	1077	1058	59.97	55.00	2.00	0.00
**Product length: 150 bp**							
Forward 2	ACCTAAGCAACGTCCAGTCA	901	920	60.12	50.00	4.00	1.00
Reverse 2	TGTGGCTCTCAATCCTCCTC	1077	1058	59.97	55.00	2.00	0.00
**Product length: 177 bp**							
Forward 3	GAGGAGGATTGAGAGCCACA	1058	1077	59.97	55.00	2.00	2.00
Reverse 3	GGAAATGTCAGGGGACTTGC	1154	1135	59.98	55.00	4.00	2.00
**Product length: 97 bp**							

b)

**House keeping: *β-actin1*** (NM_131031.1): *Danio rerio* beta-actin 1 mRNA, 1700 bp							
**Primer pair**	**Sequence (5' → 3')**	**Start**	**Stop**	**Tm**	**GC%**	**Self complementarity**	**Self 3' complementarity**
Forward	CGAGCAGGAGATGGGAACC	722	740	61.00	63.16	2.00	2.00
Reverse	CAACGGAAACGCTCATTGC	823	805	59.38	52.63	3.00	2.00
**Product length: 102 bp**							
**House keeping: *Ef1a*** (NM_131263.1): *Danio rerio* elongation factor 1a mRNA,							
Forward	CTGGAGGCCAGCTCAAACAT	678	697	61.49	55.00	5.00	2.00

Reverse	ATCAAGAAGAGTAGTACCGCTAGCATTAC	764	736	63.62	41.38	8.00	8.00
**Product length: 87 bp**							
**House keeping: *Rpl13-α*** (NM_212784.1): *Danio rerio* ribosomal protein L13alpha mRNA, 819 bp							
Forward	TCTGGAGGACTGTAAGAGGTATGC	348	371	62.37	50.00	3.00	2.00
Reverse	AGACGCACAATCTTGAGAGCAG	495	474	62.08	50.00	4.00	1.00
**Product length: 148 bp**							

Tab. S2 a) Assay primer pair design for the zebrafish genes *usp9*, *mapta* and *maptb*. After designing several primer pairs for each target gene using the NCBI Primer-BLAST tool, three different pairs of primers were selected and initially tested for specificity (by qualitative PCR) and efficiency (by quantitative PCR). All primer pairs tested displayed sufficient specificity and efficiency. Here, only the most specific and efficient pair (highlighted by the star symbol: *) was used for further experiments. b) Three zebrafish house keeping genes (*β-actin1*, *elongation factor 1* and *60S ribosomal protein L13*) were processed in parallel in all experiments.

Primer	Name	Sequence	Target
Yifor1	667_Usp9Yi_for1	CTGGGCAGCTTATGAATTGT	USP9Y
Yirev1	668_Usp9Yi_rev1	CCTCAAACTATGATTTTCTTCACC	USP9Y
Xifor1	669a_Usp9Xi_for1a	TGTTAATTCTTTGCCACTGGAGTC	USP9X
Xifor2	669b_Usp9Xi_for1b	TAATTCTTTGCCACTGGAGTC	USP9X
Xirev2	670_Usp9Xi_rev1	CTTCTCGGACAACTGCTGTCA	USP9X
XYfor1	671_Usp9XY_for1	GAACTTCTCTGGCAGGTTGC	USP9XY
XYrev1a	672a_Usp9XY_rev1a	CTGAGGATTGCCAGTATATGGTCTTC	USP9XY
XYrev1b	672b_Usp9XY_rev1b	CTGAGGATTGCCAGTATATGGTC	USP9XY
XUfor1	673_Usp9X_3utr_for1	AAAGAAACAGCCCCCAGAAT	USP9X
XUrev1	674_Usp9X_3utr_rev1	AGGGCTTAGAAACAGCAGCA	USP9X
XUfor2	675_Usp9X_3utr_for2	AAGGGCTTTTGCCCTATTGT	USP9X
XUrev2	676_Usp9X_3utr_rev2	AAAGACACAAGGCTGGGAAA	USP9X
MAPTfor	049_MAPT_for	AAGGGGGCTGATGGTAAAAC	MAPT
MAPTrev	050_MAPT_rev	CAGAGCTGGGTGGTGTCTTT	MAPT
SIRT1for	591_hSIRT1_for	TCAGTGGCTGGAACAGTGAG	SIRT1
SIRT1rev	592_hSIRT1_rev	TCTGGCATGTCCCACTATCA	SIRT1

Tab. S3 Primers tested for the knockdown experiments in the DU145 cell culture model

**Step**	**time [s]**	**temperature**	**cycles**
Initial denaturation	120	95	1
Denaturation	15	95	45
Annealing	30	60	
Elongation	60	68	
Final elongation	300	68	1

Tab. S4 PCR program used for the DU145 cell culture model PCR analyses

Description	ID	Hairpin sequence
Usp9XY	679	AAAAAGAGTGGCTGGAAGTTTGAAATTCTCTTGAAATTTCAAACTTCCAGCCACTC
Usp9X	688	AAAAAGCACTTTCCCTCCAAATTTATAGGTGTAATCTCTTGAATTACACCTATAAATTTGGAGGGAAAGTGC
scrambled	690	AAAAAGGCAGGAGTATTCGACAGTAATCTCTTGAATTACTGTCGAATACTCCTGCC
scrambled	691	AAAAAGGATCAAACAAGTGCAATAGTTCTCTTGAAACTATTGCACTTGTTTGATCC
scrambled	692	AAAAAGAGTTCTAACCGCTTATATATTCTCTTGAAATATATAAGCGGTTAGAACTC

Tab. S5 Hairpin sequences for knockdown experiments in the DU145 cell culture model

Assay ID	Catalog Number	Gene Symbol	Gene Name
Hs01126048_mH	4351372	USP9X	hCG18381 Celera Annotation;ubiquitin specific peptidase 9; X-linked
Hs01079350_mH	4351372	USP9Y	ubiquitin specific peptidase 9; Y-linked;hCG15064 Celera Annotation
Hs00902194_m1	4331182	MAPT	microtubule-associated protein tau;hCG27664 Celera Annotation
Hs03044880_gH	4331182	HSPA8	hCG2032950 Celera Annotation;heat shock 70kDa protein 8
Hs99999904_m1	4331182	PPIA	peptidylprolyl isomerase A (cyclophilin A);hCG2014648 Celera Annotation

Tab. S6 Assay IDs and catalog numbers for the TaqMan® gene expression assays used for the target and reference genes assessed by RT-PCR with the Fluidigm 48.48 integrated fluidic circuit platform for the DU145 cell culture experiments.

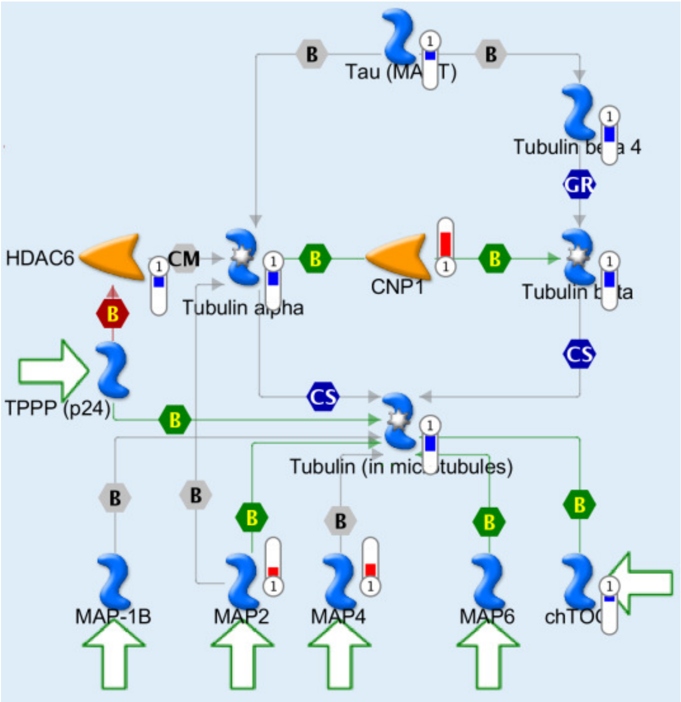

Fig. S1 Molecular sub-network for the pathway "Regulation of cytoskeleton proteins in oligodendrocyte differentiation and myelination" (from GeneGO) showing a consistent under-expression of tubulins and the microtubule-associated protein tau (MAPT) in the USP9XY and USP9X knockdowns (under-expressed genes are highlighted by blue vertical bars, over-expressed genes by red vertical bars; physical interactions represented by arrows are labeled as follows: B = general binding interaction, CS = protein complex subunit interaction, GR = interaction between groups of related biomolecules; activating interactions are highlighted in green, inhibiting interactions in red, and interactions with unspecified/unknown effects are colored in gray; white arrows with green border indicate starting points of signaling paths).

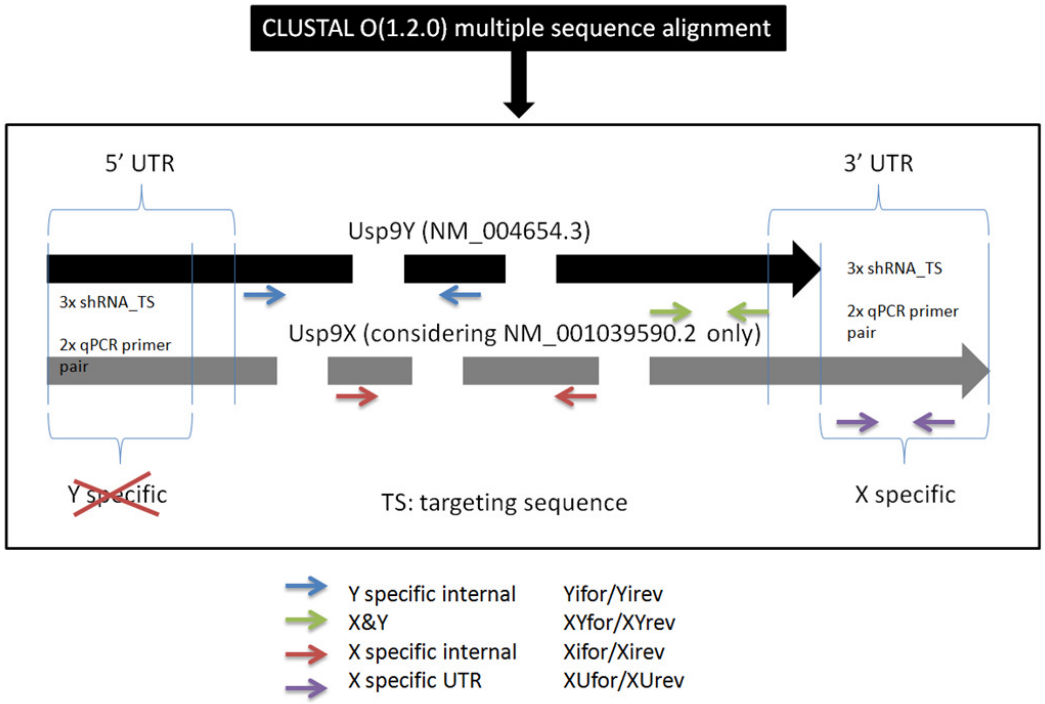

Fig. S2 Primer design scheme for the human genes USP9X and USP9Y for the DU145 cell culture experiments.

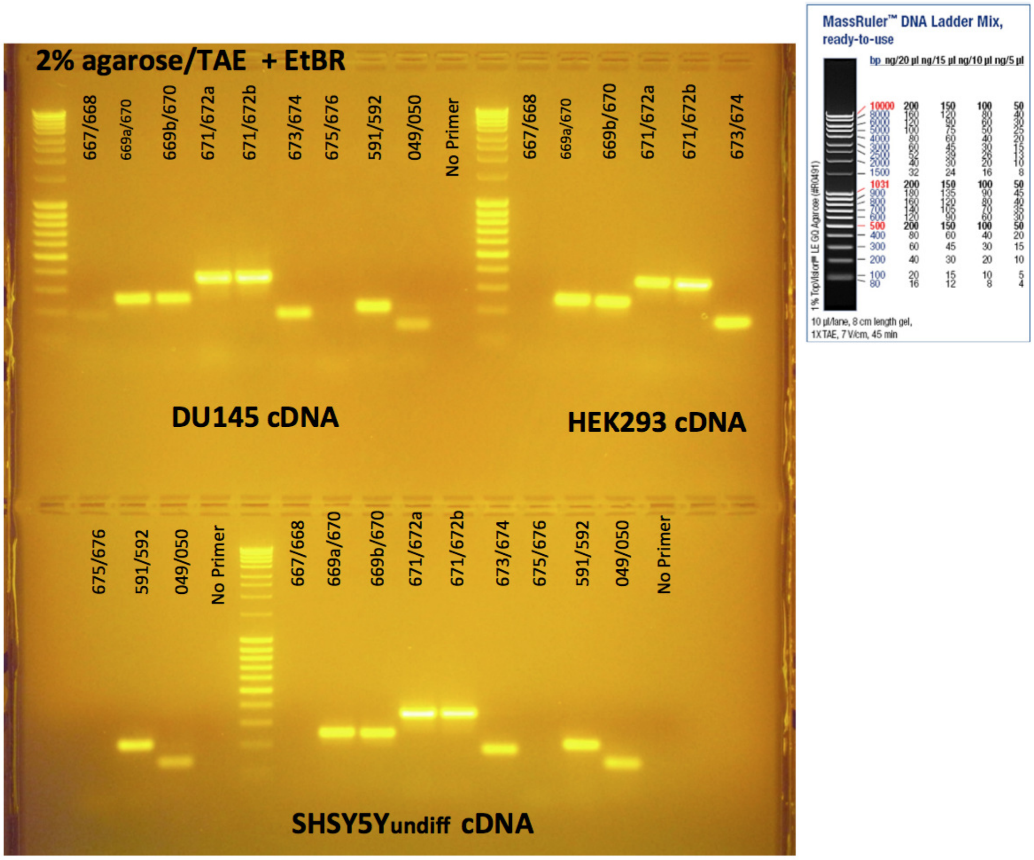

Fig. S3 Agarose gel electrophoresis results for testing primers in different cell cultures (see also the corresponding primer sequences in Suppl. Table S3, the PCR program in Suppl. Table S4, and the primer design scheme in Suppl. Fig. S2). PCR reactions in the three cell lines DU145, HEK293 and SHSY5Y are compared. As expected, USP9Y (primer pair 667/668) was detectable in the male cell line DU145 but not in the female cell lines HEK293 and SHSY5Y.
